# Response mechanisms of different *Saccharomyces cerevisiae* strains to succinic acid

**DOI:** 10.1186/s12866-024-03314-4

**Published:** 2024-05-08

**Authors:** Cai-Yun Xie, Ran-Ran Su, Bo Wu, Zhao-Yong Sun, Yue-Qin Tang

**Affiliations:** 1 College of Architecture and Environment, Sichuan University, No. 24 South Section 1 First Ring Road, Chengdu, 610065 Sichuan China; 2 Sichuan Environmental Protection Key Laboratory of Organic Wastes Valorization, No. 24 South Section 1 First Ring Road, Chengdu, 610065 Sichuan China; 3 Biogas Institute of Ministry of Agriculture, Renmin Rd. 4-13, Chengdu, 610041 Sichuan China; 4 Engineering Research Center of Alternative Energy Materials & Devices, Ministry of Education, No. 24 South Section 1 First Ring Road, Chengdu, 610065 Sichuan China

**Keywords:** *Saccharomyces cerevisiae*, Succinic acid, Genetic background, Comparative transcriptomic analysis, Response mechanism

## Abstract

**Background:**

The production of succinic acid (SA) from biomass has attracted worldwide interest. *Saccharomyces cerevisiae* is preferred for SA production due to its strong tolerance to low pH conditions, ease of genetic manipulation, and extensive application in industrial processes. However, when compared with bacterial producers, the SA titers and productivities achieved by engineered *S. cerevisiae* strains were relatively low. To develop efficient SA-producing strains, it’s necessary to clearly understand how *S. cerevisiae* cells respond to SA.

**Results:**

In this study, we cultivated five *S. cerevisiae* strains with different genetic backgrounds under different concentrations of SA. Among them, KF7 and NBRC1958 demonstrated high tolerance to SA, whereas NBRC2018 displayed the least tolerance. Therefore, these three strains were chosen to study how *S. cerevisiae* responds to SA. Under a concentration of 20 g/L SA, only a few differentially expressed genes were observed in three strains. At the higher concentration of 60 g/L SA, the response mechanisms of the three strains diverged notably. For KF7, genes involved in the glyoxylate cycle were significantly downregulated, whereas genes involved in gluconeogenesis, the pentose phosphate pathway, protein folding, and meiosis were significantly upregulated. For NBRC1958, genes related to the biosynthesis of vitamin B6, thiamin, and purine were significantly downregulated, whereas genes related to protein folding, toxin efflux, and cell wall remodeling were significantly upregulated. For NBRC2018, there was a significant upregulation of genes connected to the pentose phosphate pathway, gluconeogenesis, fatty acid utilization, and protein folding, except for the small heat shock protein gene *HSP26*. Overexpression of *HSP26* and *HSP42* notably enhanced the cell growth of NBRC1958 both in the presence and absence of SA.

**Conclusions:**

The inherent activities of small heat shock proteins, the levels of acetyl-CoA and the strains’ potential capacity to consume SA all seem to affect the responses and tolerances of *S. cerevisiae* strains to SA. These factors should be taken into consideration when choosing host strains for SA production. This study provides a theoretical basis and identifies potential host strains for the development of robust and efficient SA-producing strains.

**Supplementary Information:**

The online version contains supplementary material available at 10.1186/s12866-024-03314-4.

## Background

Succinic acid (SA), a C-4 building block chemical, has been widely used in medicine, agriculture, industry, and so on [1]. For example, succinate is widely used as an intermediary feedstock to produce chemicals such as 1,4-butanediol, tetrahydrofuran, γ-butyrolactone, succinate salts, and adipic acid [2]. In 2004, the U.S. Department of Energy (DOE) proposed that SA is one of the five most promising bio-based platform chemicals.

The production of SA via petrochemical processing is facing challenges posed by unsustainable fossil energy supplies and increased environmental burdens. Therefore, microbial factories have become a promising alternative. Microorganisms such as *Actinobacillus succinogenes* [3], *Anaerobiospirillum succiniciproducens*, *Mannheimia succiniciproducens* [4], *Corynebacterium glutamicum* [5], *Basfia succiniciproducens* [6], *Enterobacter* sp. LU1 [7] and engineered strains of *Escherichia coli* [8] have been identified as potential candidates for SA production. Generally, these bacteria require a neutral pH to ensure optimal cell growth and fermentation, however, an acidic environment is more conducive to efficient SA recovery and cost reduction. Thus, in order to develop effective SA-producing strains, the ideal microorganism should exhibit robustness, SA tolerance, and viability under low pH conditions. Notably, *Saccharomyces cerevisiae* (*S. cerevisiae*) is the favored species for industrial-scale SA production due to its superior tolerance to low pH compared to bacteria [9], and it is widely used in the industry.

Due to the inherent limitations of *S. cerevisiae* regarding SA accumulation [10], extensive efforts have been directed to enhancing the yeast’s ability to produce SA [11–15]. However, the highest SA titer achieved by *S. cerevisiae* currently stands at 43 g/L [16], notably lower than those attained by bacteria [2]. From an industrial perspective, there is a pressing demand to elevate both the SA titer and productivity in *S. cerevisiae*. However, the exact factors that limit SA production within *S. cerevisiae* remain unclear, which complicates the rational design of a robust cell factory. Generally, product toxicity is recognized as one of the major bottlenecks for SA production. To overcome this, it is necessary to gain a better understanding of the physiological effects of SA and how *S. cerevisiae* cells respond to SA stress. However, no relevant studies have been published on this subject so far.

Considering the potential variability in *S. cerevisiae* strains’ response to SA, we compared the tolerances of five distinct *S. cerevisiae* strains toward SA stress (Table 1). Among these, Kagoshima 5 is used for brewing the Japanese distilled spirit shochu, while A1 is usually used for the industrial ethanol production. KF7 is well studied for ethanol production and stress tolerance in our previous studies [17]. These strains were firstly compared for their growth under different concentrations of SA. Three representative strains were chosen to explore their transcriptomic responses to different concentrations of SA. Given that the protein quality control (PQC) was involved in the response to SA across all three strains, we validated the role of PQC-related genes in SA tolerance. This study revealed that intracellular acetyl-CoA levels, protein folding activity, and the integrity of the cell wall and membrane are vital for the response and tolerance of *S. cerevisiae* to SA stress. These findings provide promising modification targets for the subsequent development of robust and efficient industrial SA-producing *S. cerevisiae* strains.

**Table 1:** Saccharomyces cerevisiae strains and plasmids used in this study

Strains	Genotype	Origin
KF7	*MATa/α*, *Flo*^*+*^	[18]
NBRC1958	*MATa/α*, *Flo*^*+*^	NBRC collection
NBRC2018	*MATa/α*, *Flo*^*+*^	NBRC collection
Kagoshima 5	*MATa/α*, *Flo*^*−*^	Syochu yeast
A1	*MATa/α*, *Flo*^*−*^	Angel yeast
KF7Cas9	KF7, Cas9-NAT	This study
N19Cas9	NBRC1958, Cas9-NAT	This study
N20Cas9	NBRC2018, Cas9-NAT	This study
KF7 + TSP26	KF7, Δ*yjl043w*:: *P*_*TEF1*_-*HSP26*	This study
KF7 + TSP42	KF7, Δ*yjl043w*:: *P*_*TEF1*_-*HSP42*	This study
N19 + TSP26	NBRC1958, Δ*yjl043w*:: *P*_*TEF1*_-*HSP26*	This study
N19 + TSP42	NBRC1958, Δ*yjl043w*:: *P*_*TEF1*_-*HSP42*	This study
N20 + TSP26	NBRC2018, Δ*yjl043w*:: *P*_*TEF1*_-*HSP26*	This study
N20 + TSP42	NBRC2018, Δ*yjl043w*:: *P*_*TEF1*_-*HSP42*	This study
Plasmids		
pMEL13	2 μm, *ampR*, *KanMX*, *gRNA-CAN1.Y*	[19]
Cas9-NAT	*ampR*, *NAT*, *Cas9*	[19]
pM-gYJL043W	pMEL13, *gRNA-YJL043W*	This study
pM-gYJL043W-N20	pMEL13, *gRNA-YJL043W* (NBRC2018)	This study
19T-HSP26	*P*_*HSP26*_-*HSP26-T*_*HSP26*_	This study
19T-HSP42	*P*_*HSP42*_-*HSP42-T*_*HSP42*_	This study
19T-TSP26	*P*_*TEF1*_-*HSP26-T*_*HSP26*_	This study
19T-TSP42	*P*_*TEF1*_-*HSP42-T*_*HSP42*_	This study

Flo: Flocculation

## Methods

### Strains and media

All strains used and constructed in this study were listed in Table 1. Five diploid *S. cerevisiae* strains were tested for their tolerances to SA, including KF7 [18], NBRC1958 (also known as NCYC625), NBRC2018 (NBRC collection), Kagoshima 5 (a Syochu yeast), and A1 (Angel yeast). Among these, KF7, NBRC1958, and NBRC2018 were used as the parental strains for genetic manipulation. *E. coli* DH5α (Takara Bio Inc., Japan) was used for gene cloning and manipulation.

YPD medium (10 g/L yeast extract, 20 g/L peptone, 20 g/L glucose) was used for the cultivation of yeast cells. YPD agar plates supplemented with 100 µg/mL geneticin, 50 µg/mL nourseothricin, or both, were used to select yeast transformants. To assess the SA tolerance of the strains, YPD medium was supplemented with SA at final concentrations of 20 g/L, 60 g/L and 80 g/L, respectively. Since the addition of SA lowered the pH of the YPD medium to around 3.0, all YPD media (with or without SA) were adjusted to pH 3.0 using a HCl solution to eliminate the effect of pH fluctuations on cell growth. Luria-Bertani (LB) medium (5 g/L yeast extract, 10 g/L peptone, 10 g/L NaCl, pH 7.0) supplemented with 100 µg/mL ampicillin, 100 µg/mL kanamycin, or 50 µg/mL nourseothricin was used to select *E. coli* transformants.

### Succinic acid-tolerance test

Yeast cells were initially inoculated into 3 mL of YPD medium and cultured overnight at 30˚C and 200 rpm. Then, the cells were harvested and inoculated into 50 mL shake flasks containing 20 mL of YPD medium with different concentrations of SA. Cultures were further incubated for 24 h at 30˚C and 200 rpm. During the cultivation process, broth samples were periodically taken to analyze the concentration of cells. Three replicated cultivation experiments were independently performed. The cell growth was monitored spectrophotometrically by measuring the absorbance at 600 nm (OD_600_) periodically. For the flocculating strains, cells were dispersed using 0.1% (w/v) EDTA prior to taking the OD_600_ measurements. Statistical significance was evaluated using the unpaired Student’s *t*-test. The level of statistical significance was set to *p* < 0.05.

### RNA isolation

At the 6 h of cultivation, which typically corresponds to the early exponential growth phase, 0.5 mL of yeast culture containing approximately 2 × 10^7 cells were collected. Total RNA was isolated from cells using the Yeast RNA Kit (Omega Biotek, USA) according to the manufacturer’s instructions. RNA quality was determined by performing agarose gel electrophoresis. The RNA concentration was measured by using a NanoDrop 2000 spectrometer (Life Technology, USA). Each RNA sample used for microarray analysis consisted of a mixture of three independent biological replicates.

### Microarray analysis

Microarray analysis was performed using the 7G Affymetrix GeneChip® Yeast Genome 2.0 Array, according to the method described previously [20]. Data extraction and analysis were performed using the Affymetrix GeneChip Command Console Software. The microarray data can be accessed through the GEO accession GSE193190. The gene annotation information was sourced from the Saccharomyces Genome Database (SGD, http://www.yeastgenome.org). Differentially expressed genes (DEGs) were identified using the combined criteria of |fold change (FC) |≥ 2 and *p* value < 0.05. KEGG pathways were retrieved from the KEGG database (http://www.kegg.jp/kegg) and analyzed using the Metascape web server (https://metascape.org) with significant cut-off values of *p* < 0.005. Protein-protein interaction (PPI) analysis was also performed using the Metascape web server.

### Plasmid construction

The plasmids and primers used in this study were summarized in Tables 1 and 2. Plasmids 19T-HSP26 and 19T-HSP42 with native promoter were constructed as follows: The DNA sequences encoding the *HSP26* and *HSP42* genes were amplified from the genomic DNA of KF7 by PCR using the YJL-H26-F/R and YJL-H42-F/R primer sets, respectively. The resulting PCR products were cloned into pMD19 vector separately to build plasmids 19T-HSP26 and 19T-HSP42. Considering that the *TEF1* gene displays high and stable expression levels under different SA conditions (Table S1), its promoter was used for the overexpression of the *HSP26* and *HSP42* genes. Plasmids 19T-TSP26 and 19T-TSP42 with the *TEF1* promoter were constructed as follows: The DNA fragments containing the *TEF1* promoter region (Table S2) were amplified from KF7 genomic DNA by PCR using the YJL-TEF1 proF and H26-TEF1 proR or H42-TEF1 proR primers, respectively. Likewise, the corresponding vector fragments were amplified from 19T-HSP26 and 19T-HSP42 using the TEF1p-YJL R and TEF1p-H26 F or TEF1p-H42 F primers, respectively. These fragments were connected using Gibson assembly [21]. The resulting plasmids were named as 19T-TSP26 and 19T-TSP42, respectively.

**Table 2:** Primers used in this study

Primers	Sequences (5’-3)
Construction of 19T-HSP26 and 19T-HSP42	
YJL-H42-F	CAGGAAAACCGCAAGCCATGTTTGTTGATTATTCCGGACTGGAGCGTTAAGCTGGGGTTGGGTAACAAGTG
YJL-H42-R	AACAACGGGTCCGTAGTAGGTAGCGCACCCAACAGTGCCTCCAACTGCACACCTCTTTTGGTGGGCTGAG
YJL-H26-F	CAGGAAAACCGCAAGCCATGTTTGTTGATTATTCCGGACTGGAGCGTTACGTGATTCTCGCTCGGAATCCGTC
YJL-H26-R	AACAACGGGTCCGTAGTAGGTAGCGCACCCAACAGTGCCTCCAACTGCACCGGTCATATATCGAAGCCAAAGC
Construction of 19T-TSP26 and 19T-TSP42	
YJL-TEF1 proF	TATTCCGGACTGGAGCGTTACAGAAAGCGACCACCCAACT
H42-TEF1 proR	GGTTGATAAAAACTCATTTTGTAATTAAAACTTAGATTAGATTGC
H26-TEF1 proR	GGACTGTTAAATGACATTTTGTAATTAAAACTTAGATTAGATTGC
TEF1p-H42 F	GCAATCTAATCTAAGTTTTAATTACAAAATGAGTTTTTATCAACC
TEF1p-H26 F	GCAATCTAATCTAAGTTTTAATTACAAAATGTCATTTAACAGTCC
TEF1p-YJL R	AGTTGGGTGGTCGCTTTCTGTAACGCTCCAGTCCGGAATA
Amplification of repair fragments	
SA-F	CAGGAAAACCGCAAGCCATGT
SA-R	AACAACGGGTCCGTAGTAGGTAG
N20HR-F	CAGGAAAACCGCAAGCCATGGTTGTTGGTTATTCCGGACTGGAGCGTTA
N20HR-R	AGGACGGGTGAACATCGAACGGGGCCGACGGCCTAAAAGAGAAGCTGATGAACAGTGCCTCCAACTGCAC
Transformants verification	
Dg-YJL-MT7-F	ACCGCTTACAGGCCTAAACC
Dg-YJL-MT7-R	TGGCCTTGGACTCGGATTTC
M13-47	CGCCAGGGTTTTCCCAGTCACGAC
RV-M	GAGCGGATAACAATTTCACACAGG
Construction of gRNA plasmid	
gYJL043W F	P-**CCTGTGTGTTTTACCGTTGA**GTTTTAGAGCTAGAA
N20-gYJL043W F	P-**ATCATCCCCATGCTGTTTAT**GTTTTAGAGCTAGAA
gRNA-R	P-GATCATTTATCTTTCACTGCGGAGAAG
pMseq1	ACTTGATGTTTTCTTTCGAG

N20: strain NBRC2018; the bold 20 bases are target sequences of guide RNA targeting *YJL043W* loci; “underline” indicates homologous arm; 5’P: 5’-Phosphate modification

To construct a guide RNA (gRNA) plasmid, it was necessary to find an appropriate gene-insertion site. Based on the observation that the expression level of the *YJL043W* gene is quite low and not show sensitivity to SA (Table S1), the *YJL043W* loci can be used for gene integration. A specific guide RNA sequence was designed to target the *YJL043W* gene by using the Yeastriction tool [19]. It should be note that the *YJL043W* gene sequences are identical in NBRC1958 and KF7, while there is a slight variation in NBRC2018. Thus, two sets of primers were prepared: primer gYJL043W F, which contains the gRNA sequences (CCTGTGTGTTTTACCGTTGA) targeted to the *YJL043W* gene in KF7 and NBRC1958, and primer N20-gYJL043W F, which contains the gRNA sequences (ATCATCCCCATGCTGTTTAT) targeted to *YJL043W* gene in NBRC2018. To construct the gRNA plasmids, the linearized plasmid backbones were amplified from the plasmid pMEL13 using the phosphorylated primers gRNA-R and gYJL043W F or N20-gYJL043W F. These two PCR products were self-linked individually to form the plasmids pM-gYJL043W and pM-gYJL043W-N20. All the sequences were confirmed by sequencing.

### Strain construction

Genes *HSP26* and *HSP42*, which encode the only two small heat shock proteins (sHsps) in *S. cerevisiae*, were individually overexpressed using CRISPR/Cas9 editing system. Yeast transformation was carried out by using the lithium acetate method as described previously. Firstly, strains KF7, NBRC1958, and NBRC2018 were transformed with the Cas9-NAT [19] plasmid to generate strains KF7Cas9, N19Cas9, and N20Cas9, respectively. Then, the repair fragments were amplified with primers SA-F and SA-R (for KF7 and NBRC1958), as well as N20HR-F and N20HR-R (for NBRC2018) using plasmids 19T-TSP26 and 19T-TSP42 as template, respectively. The repair fragments and gRNA plasmids were then transformed into the corresponding strains already harboring the Cas9-NAT plasmid. Transformants were selected on YPD plates containing geneticin and nourseothricin, and further confirmed by colony PCR and Sanger sequencing. The correct transformants were cultured in YPD media to remove the Cas9-NAT and gRNA plasmids according to Mans’ method [19]. The transformants were subjected to the SA tolerance test.

## Results and discussion

### Succinic acid tolerance

**Fig. 1:**
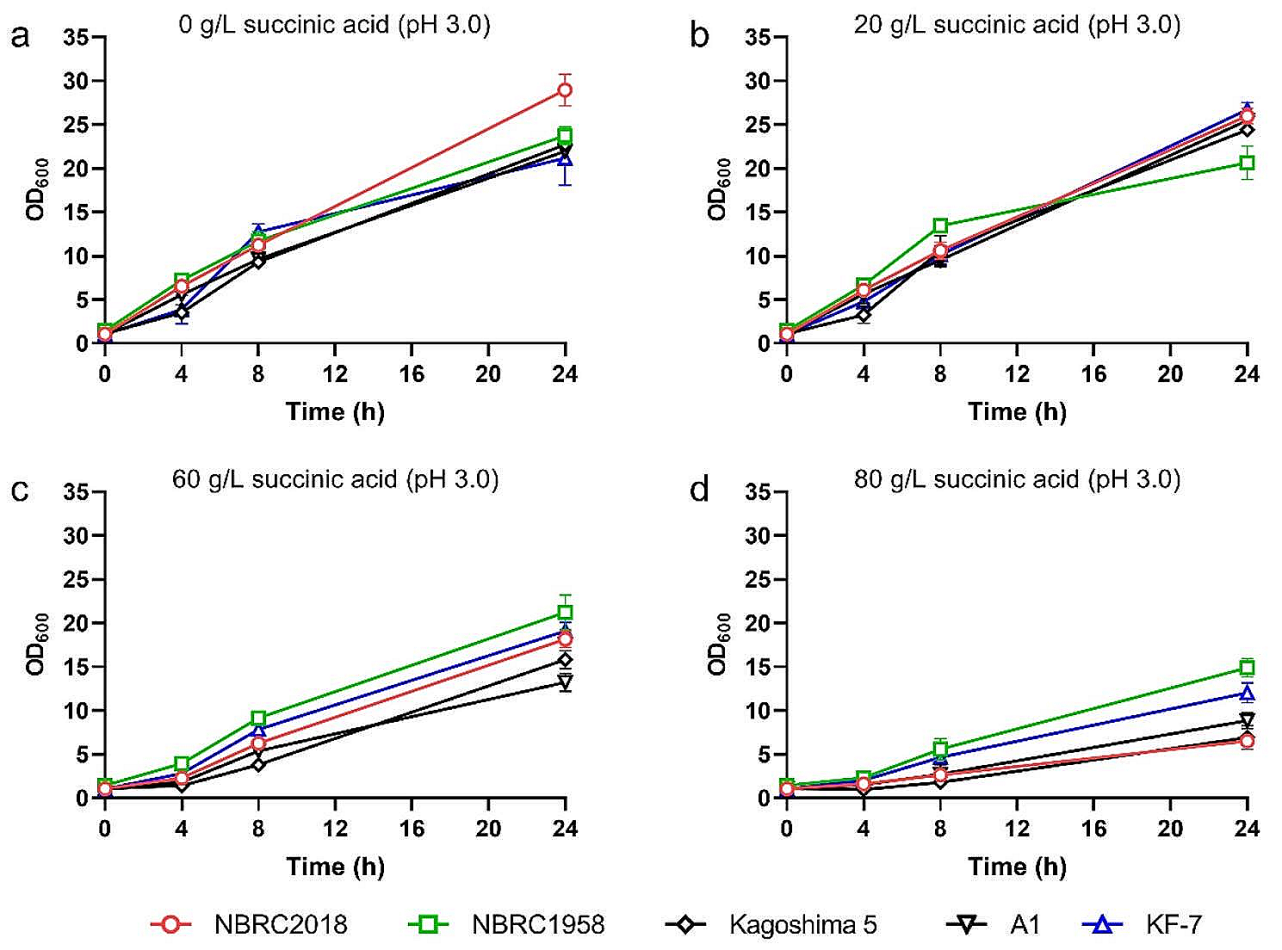
Growth of *S. cerevisiae* strains NBRC2018 (circle), NBRC1958 (square), Kagoshima 5 (diamond), A1 (inverted triangle), and KF7 (triangle) under (a) 0 g/L, (b) 20 g/L, (c) 60 g/L, and (d) 80 g/L SA, respectively. The initial inoculum was OD_600_ 1.0. Data were shown as the mean ± standard error (*n* = 3)

Five strains were evaluated under different concentrations of SA (Fig. 1), and the growth inhibition ratios at 24 h were displayed in Table 3. Under the condition of 20 g/L SA, the growth of NBRC1958 and NBRC2018 was inhibited, whereas the growth of Kagoshima 5, A1, and KF7 was promoted. Upon exposure to 60 g/L SA, all five strains exhibited varying degrees of growth inhibition. The inhibitory effect became more pronounced when the SA concentration increased to 80 g/L SA.

**Table 3:** The growth inhibition ratios (%) of five strains under different concentrations of SA

SA	20 g/L	60 g/L	80 g/L
KF7	-26.21	9.81	43.10
NBRC1958	12.95	10.53	37.23
NBRC2018	10.31	37.29	77.46
A1	-16.05	39.80	59.73
Kagoshima 5	-7.53	30.29	69.46

The growth inhibition ratio = (OD_600_ without SA - OD_600_ with SA)/ OD_600_ without SA. Positive values represent growth inhibition, while negative values represent growth promotion. The initial inoculum was OD_600_ 1.0. Data were calculated at 24 h of cultivation. Average values were calculated from three replicates

KF7 and NBRC1958 showed relatively low inhibition ratios at 60 g/L and 80 g/L SA, indicating good tolerances to SA. Both of them may be promising platforms for SA production. However, the growth of KF7 was promoted by 20 g/L SA, whereas the growth of NBRC1958 was inhibited. This observation suggests that the two strains might adopt different mechanisms to respond to SA, especially at 20 g/L SA. In contrast, NBRC2018 showed the highest growth inhibition ratio at different SA concentrations, which implied that it is not a good candidate for SA production due to its poor resistance to SA. In order to systematically understand the response mechanism of *S. cerevisiae* to SA, KF7, NBRC1958, and NBRC2018 were selected as three representative strains for comparative transcriptomic analysis.

### Transcriptional response of KF7 to different concentrations of SA

**Fig. 2:**
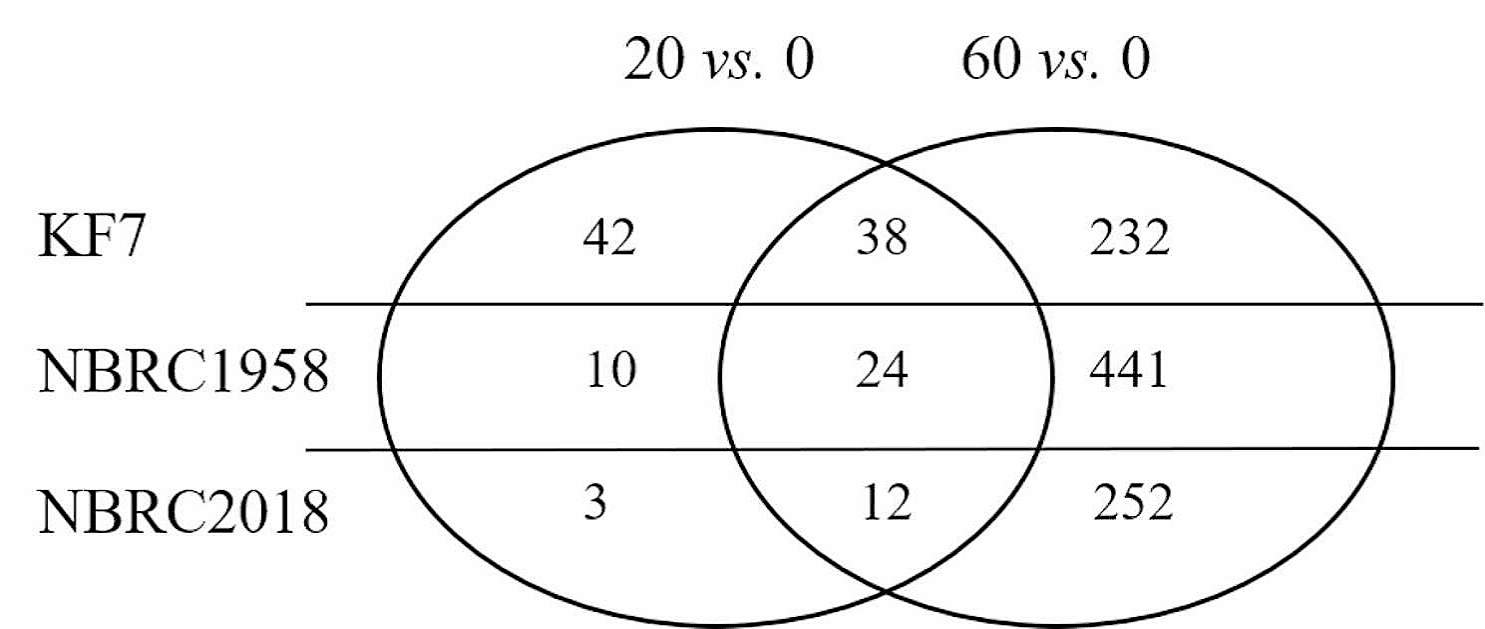
Venn grams of differentially expressed genes of KF7, NBRC1958, and NBRC2018 under different concentrations of SA. 20 vs. 0 and 60 vs. 0 indicated comparisons 20 vs. 0 and 60 vs. 0, respectively

**Fig. 3:**
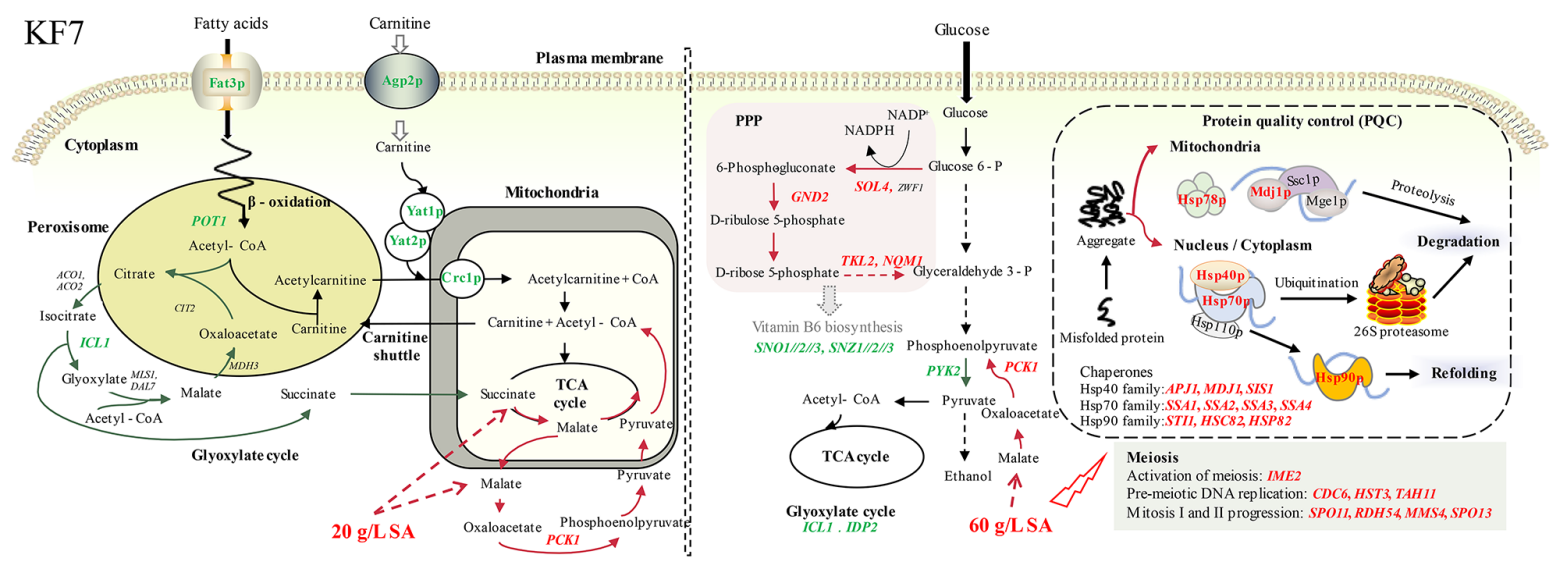
Illustration of the response mechanisms of *S. cerevisiae* strains KF7 to SA at different concentrations. Red text indicated upregulation and green text indicated downregulation

When comparing the 20 g/L SA group with the control group (referred to as 20 vs. 0), 80 DEGs were identified (Fig. 2). Based on the KEGG enrichment analysis, peroxisome was significantly enriched (Table S3). Protein-protein interaction (PPI) analysis also indicated the importance of peroxisome (Fig. S1a). Thus, changes in peroxisome might be the key response of cells to 20 g/L SA (Fig. 3).

Genes (*POT1*, *YAT1*, and *YAT2*) involved in peroxisome (Table S3) are closely related to beta-oxidation of fatty acids and significantly downregulated under 20 g/L SA. *POT1* encodes acetyl-coenzyme A (CoA) C-acyltransferase, which is involved in fatty acid beta-oxidation. In *S. cerevisiae*, acetyl-CoA derived from beta-oxidation of fatty acids can be transferred to mitochondria for energy supply through two pathways: the glyoxylate cycle and carnitine shuttle [22]. The downregulation of the isocitrate lyase gene *ICL1* and the carnitine acetyltransferase genes *YAT1* and *YAT2* indicates a decrease in the transfer of acetyl-CoA from peroxisome to mitochondria. This inference is also supported by the downregulation of genes related to fatty acid uptake (*FAT3*) and carnitine transport (*CRC1* and *AGP2*). It seems that the artificial supplement of 20 g/L SA would reduce the dependence on peroxisome-derived acetyl-CoA and replenish the tricarboxylic acid (TCA) cycle with C2 units in mitochondria by driving acetyl-CoA synthesis [23, 24]. The increased acetyl-CoA in the cytoplasm may be responsible for the enhanced cell growth of KF7 under 20 g/L SA.

When comparing the 60 g/L SA group with the control group (referred to as 60 vs. 0), 270 DEGs were identified (Fig. 2). Nine pathways related to carbon metabolism, amino acid metabolism, and vitamin B6 metabolism were significantly enriched through the KEGG enrichment analysis (Table S3). Genes that participate in protein folding, meiosis, VB6, and amino acid metabolic process were also noted through PPI analysis (Fig. S1b, Table S4). Overall, KF7 adopted complex and systematic regulatory mechanisms in response to 60 g/L SA (Fig. 3).

Genes related to carbon metabolisms, such as glyoxylate and dicarboxylate metabolism, pentose phosphate pathway (PPP), and pyruvate metabolism, were significantly regulated (Table S3). The glyoxylate cycle gene *ICL1* was downregulated, and the gluconeogenesis gene *PCK1* was upregulated in response to 60 g/L SA. In yeast, the glyoxylate cycle converts (iso)citrate and acetyl-CoA to succinate and malate, which are the precursors for gluconeogenesis [25, 26]. It was speculated that the excess exogenous SA might inhibit succinate formation in the glyoxylate cycle, meanwhile, promote gluconeogenesis.

Several key genes in the PPP were significantly upregulated, including *SOL4* and *GND2* in the oxidative PPP branch, as well as *TKL2* and *NQM1* in the non-oxidative PPP branch. PPP plays a pivotal role in the oxidative stress response. It generates the primary redox factor NADPH for the antioxidative machinery [27, 28]. Furthermore, PPP functions as a metabolic redox sensor and regulator [29]. It has been reported that an increased PPP flux is accompanied by a reduced glycolytic flux [30], which explains the downregulation of the glycolysis gene *PYK2* in KF7.

Genes (*SSA1*, *SSA2*, *SSA3*, *SSA4*, *APJ1*, *HSP78*, *SIS1*, *HSC82*, *HSP82*, *STI1*, and *MDJ1*) involved in protein folding were all upregulated in response to 60 g/L SA (Fig. S1b, Table S4). The proteins encoded by these genes play essential roles in resolving misfolded proteins within the protein quality control (PQC) network [31, 32]. As reported in previous studies, the induced protein chaperones (encoded by *SSA1*, *SSA2*, *SSA3*, and *SSA4*) provide adaptive responses to intracellular stress [33]. Meanwhile, the high PQC activity induced by mild stress has been proven to enhance the tolerance of *S. cerevisiae* cells to severe ethanol stress [34]. Therefore, we hypothesized that the upregulation of PQC genes might contribute to cell survival under 60 g/L SA stress.

Meiotic genes (*MMS4*, *IME2*, *CDC6*, *SPO11*, *HST3*, *SPO13*, *RDH54*, and *TAH11*) were significantly upregulated (Fig. S1b, Table S4). *IME2* encodes a kinase that functions as a positive regulator for pre-meiotic DNA replication and nuclear division [35]; *SPO11* encodes a meiosis-specific protein required for catalyzing DNA double-strand breaks (DSB) during meiotic recombination [36]; and *SPO13* regulates the separation of homologous chromosomes at meiosis I [37]. The result indicated that sporulation is one of the ways that KF7 responds to a high concentration of SA. This phenomenon was also found when *S. cerevisiae* adapts to oxidative, osmotic stress, and nutrient starvation [33].

Overall, KF7 employs different regulatory mechanisms in response to varying SA concentrations. At a low concentration of 20 g/L SA, the cell growth of KF7 was promoted, probably due to the elevated levels of intracellular acetyl-CoA. However, when faced with a higher concentration of 60 g/L SA, KF7’s cell growth was repressed. Under high SA stress, KF7 employs a complex strategy that involves suppressing the endogenous succinate formation, redirecting metabolic flux towards gluconeogenesis and the PPP, preserving protein quality, and possibly initiating sporulation.

### Transcriptional response of NBRC1958 to different concentrations of SA

**Fig. 4:**
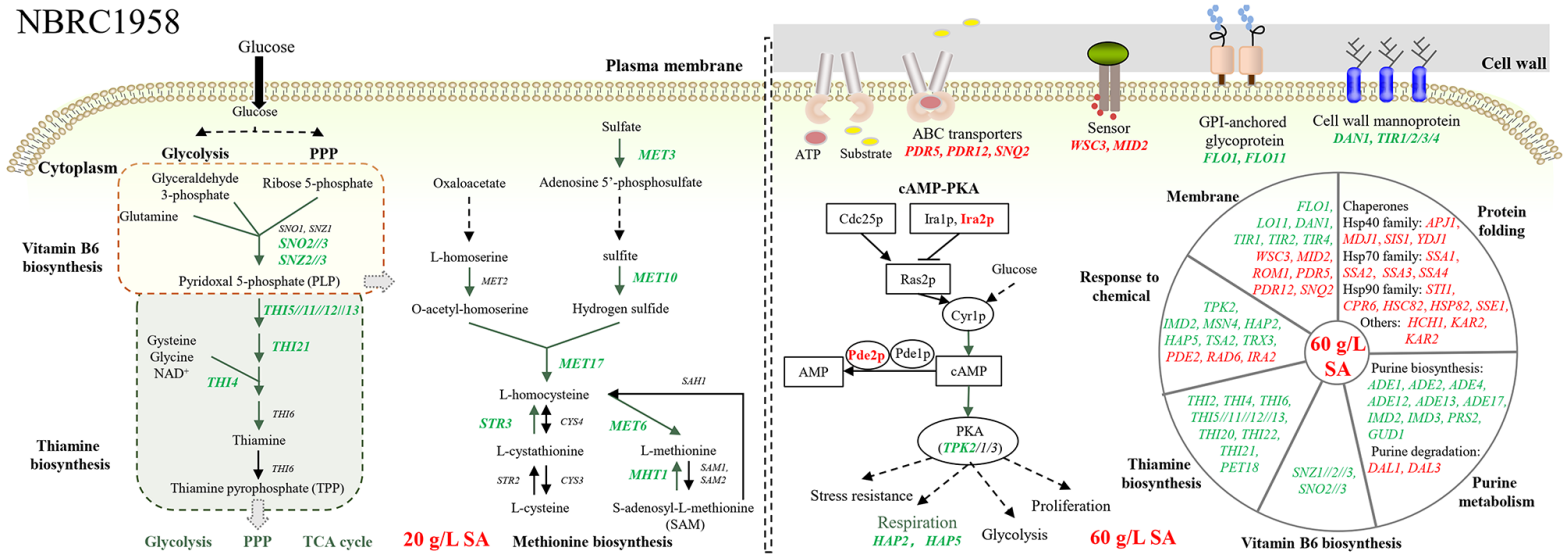
Illustration of the response mechanisms of *S. cerevisiae* strains NBRC1958 to SA at different concentrations. Red text indicated upregulation and green text indicated downregulation

A total of 34 DEGs were identified in comparison 20 vs. 0 (Fig. 2). Through the KEGG enrichment analysis, three pathways were enriched, including thiamine metabolism, vitamin B6 metabolism, and metabolic pathways (Table S3). Moreover, PPI analysis indicated the potential roles of pyridoxine and methionine in response to 20 g/L SA (Fig. S2a). These results highlight three main aspects of the cellular response to 20 g/L SA: the regulation of thiamine, vitamin B6, and methionine metabolism (Fig. 4).

Genes involved in thiamine metabolism (*THI5*//*11//12//13*, *THI4*, and *THI21*) and vitamin B6 metabolism (*SNO2*, *SNO3*, *SNZ2*, and *SNZ3*) play crucial roles in the biosynthesis of vitamin B1 and B6 [38]. The downregulation of these genes might lead to a decline in the biosynthesis of vitamin B6 and thiamine. Pyridoxal 5’-phosphate (PLP), the active form of vitamin B6, serves as a cofactor for numerous enzymes that are crucial for amino acid metabolism, glucose metabolism, and even thiamine biosynthesis [39]. Thiamine and its biologically active form, thiamine pyrophosphate (TPP), are essential cofactors in metabolic pathway, including glycolysis, the PPP, and the TCA cycle, which are all essential for cell survival [39–41]. Thus, it is reasonable to postulate that decreased levels of vitamin B6 and thiamine might lead to a reduction in metabolic activity, and thus impairing cell growth. This hypothesis is supported by earlier research [41, 42]. Additionally, genes involved in methionine biosynthesis (*MET3*, *MET10*, *MET17*, *MET6*, *MHT1*, *STR3*) were also downregulated significantly. *STR3* and *MET17* encode PLP-dependent enzymes, and their down-regulation might be due to vitamin B6 deficiency. It is noteworthy that a disruption in methionine synthesis can be lethal for fungal growth [43, 44]. Altogether, the cell growth inhibition under 20 g/L SA could be attributed to the repression of the biosynthesis of vitamin B6, thiamine, and methionine.

When it comes to comparison 60 vs. 0, 465 genes were notably regulated (Fig. 2). Through the KEGG enrichment analysis, six pathways were enriched, including thiamine metabolism, glyoxylate and dicarboxylate metabolism, and purine metabolism (Table S3). Additionally, the DEGs were classified into seven groups through PPI analysis. These categories included response to chemical, protein folding, nicotinate and nicotinamide metabolism, monosaccharide transmembrane transporter activity, etc. (Fig. S2b, Table S5). These results demonstrated that NBRC1958 employs a complex mechanism in response to 60 g/L SA (Fig. 4).

Consistent with the observations under 20 g/L SA, genes involved in thiamine and vitamin B6 metabolism continued to exhibit significant downregulated in NBRC1958 upon exposure to 60 g/L SA. Besides, purine metabolism was also affected under 60 g/L SA. Key genes in the de novo purine biosynthesis pathway (*ADE1*, *ADE2*, *ADE4*, *ADE12*, *ADE13*, *ADE17*, *IMD2*, *IMD3*, *PRS2*, and *GUD1*) and sulfate assimilation (*MET3* and *MET14*) were downregulated. On the contrary, genes responsible for purine degradation (*DAL1*, and *DAL3*) were upregulated. This result suggested a decrease in the intracellular availability of purines. Previous studies have shown that overexpression of genes related to de novo purine biosynthesis can boost cell growth and ethanol productivity in *S. cerevisiae* under various stress conditions, such as high ethanol concentration, high temperature, hydrogen peroxide exposure, and the presence of lignocellulosic biomass-derived inhibitors [45, 46]. Therefore, it is possible that the 60 g/L SA impacts the growth of NBRC1958 by altering the intracellular levels of thiamine, vitamin B6, and purine.

Several DEGs (*PDE2*, *TPK2*, *IRA2*, *HAP2*, *HAP*5, *TSA2*, *TRX3*, and *RAD6*) are related to the response to chemical (Fig. S2b, Table S5). *PDE2* encodes a cyclic AMP (cAMP) phosphodiesterase and *TPK2* encodes a cAMP-dependent protein kinase, both of which are integral parts of the cAMP-protein kinase A (PKA) signaling pathway. *IRA2* encodes a GTPase-activating protein that negatively regulates Ras2p, leading to a decrease in cAMP levels. The upregulation of *PDE2* and *IRA2*, as well as the downregulation of *TPK2*, indicates a reduction in cAMP levels and thereby, the inhibition of PKA activity. This inactivation of PKA has been known to cause yeast cells to halt proliferation and transition into the stationary phase, thereby acquiring increased stress resistance [47]. Hence, it can be deduced that cells might cease proliferation to retain viability when exposed to high SA. Besides, genes that encode chaperones promoting protein folding and facilitating the degradation of misfolded protein were also upregulated in response to 60 g/L SA. This upregulation could support cell survival as discussed in comparison 60 vs. 0 of KF7.

Several genes associated with the cell wall integrity (CWI) pathway (*WSC3*, *MID2*, and *ROM1*) were significantly upregulated. *WSC3* and *MID2* encode cell surface sensors, while *ROM1* encodes a guanine nucleotide exchange factor [48, 49]. This suggests that the strain enhanced its monitoring and response to cell wall perturbations under high concentration of SA. Moreover, genes encoding ATP-binding cassette (ABC) transporters, including *PDR5*, *PDR12*, and *SNQ2*, were also upregulated. ABC transporters are key components of the pleiotropic drug resistance (PDR) pathway that export toxic compounds from the cell [50, 51]. It’s worth noting that *PDR12* has been shown to be highly induced under low pH conditions and in the presence of weak acids [51]. This upregulation could suggest an increased effort by the cells to extrude SA. On the other hand, the cell wall mannoprotein genes (*DAN1*, *TIR1*, *TIR2*, and *TIR4*) and the flocculin genes (*FLO1* and *FLO11*) were significantly downregulated. Changes in the expression of DAN/TIR proteins have been implicated in altering the fluidity of the plasma membrane [52]. The downregulation of these genes under high SA stress may indicate that *S. cerevisiae* undergoes cell wall remodeling to change the cell surface properties.

In summary, when NBRC1958 was subjected to 20 g/L SA, the biosynthesis of vitamin B6, thiamine, and methionine was notably suppressed, which could lead to a decrease in metabolic activity and subsequent impairment of cell growth. When the SA concentration was increased to 60 g/L, the severity of growth inhibition did not intensify. This could be explained by the observation that, despite the biosynthesis of vitamin B6, thiamine, and purine remained suppressed, the cell’s defense system was activated. It potentially protects the cells from SA stress by entering stationary phase, enhancing protein folding activity, restructuring cell walls, and promoting toxin elimination.

### Transcriptional response of NBRC2018 to different concentrations of SA

**Fig. 5:**
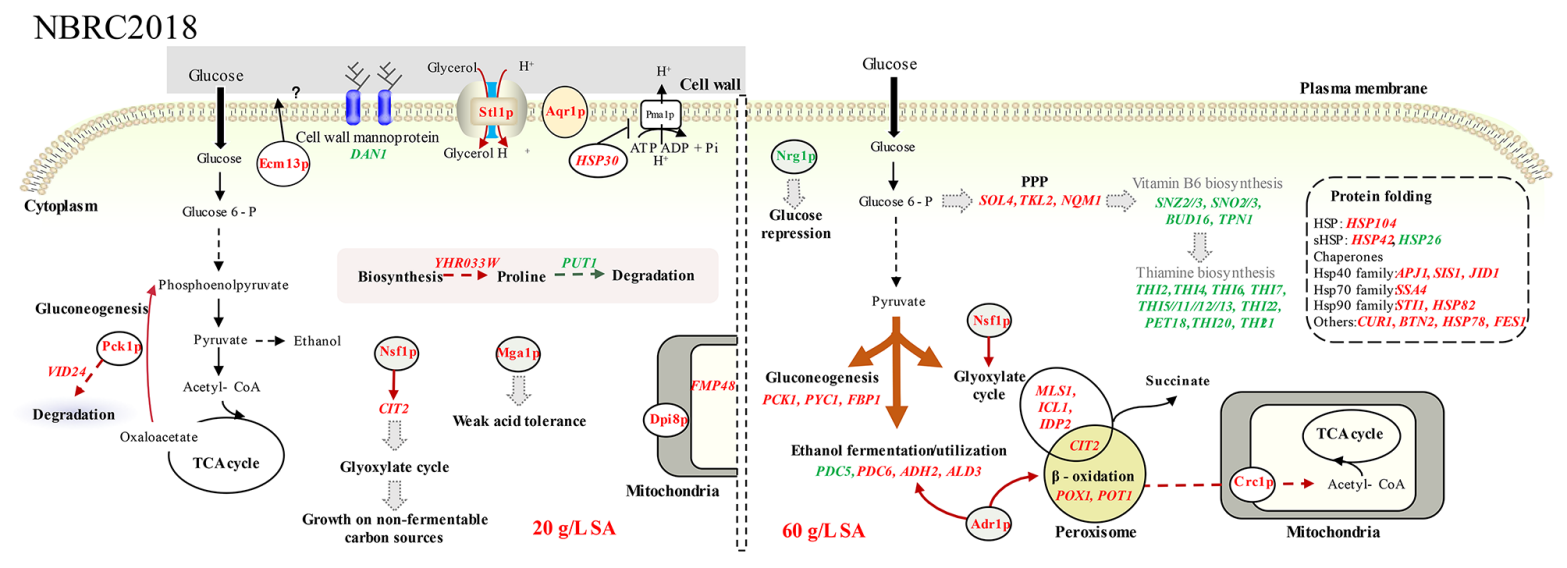
Illustration of the response mechanisms of *S. cerevisiae* strains NBRC2018 to SA at different concentrations. Red text indicated upregulation and green text indicated downregulation

Since NBRC2018 displays a weaker tolerance to SA compared to KF7 and NBRC1958, we attempted to identify the specific genes that might be contributing to the difference in SA tolerance. Only 15 DEGs were identified in comparison 20 vs. 0 (Fig. 2). No valid information was provided by KEGG enrichment analysis and PPI analysis. Functional annotation of these DEGs offered valuable insights into the transcriptional response of NBRC2018 to 20 g/L SA (Fig. 5).

Genes *PCK1* and *VID24* were significantly upregulated in response to 20 g/L SA. *PCK1* encodes phosphoenolpyruvate carboxykinase, a key enzyme that initiates gluconeogenesis. Meanwhile, *VID24*, encoding a glucose-induced degradation (GID) complex subunit, participates in the ubiquitin-mediated degradation of gluconeogenic enzymes such as Fbp1p, Pck1p, and Mdh2p [53]. Thus, we speculated that exogenous SA might induce gluconeogenesis in NBRC2018, yet the effect is subsequently attenuated by the glucose-induced degradation of gluconeogenic enzymes.

Six genes (*MGA1*, *ECM13*, *DAN1*, *STL1*, *HSP30*, and *AQR1*) related to stress resistance were significantly regulated. Our previous study highlighted the transcription factor Mga1p’s role in regulating tolerance to acetic acid and furfural [54]. Moreover, the other five genes are closely associated with the cell surface. *ECM13*, which encodes a protein of unknown function, is upregulated in response to cell wall damage and is implicated in cell wall biosynthesis and organization [55]. *HSP30* encodes a heat shock protein that negatively regulates the plasma membrane H^+^-ATPase Pma1p. Under conditions of high temperature, high ethanol exposure, high osmolarity, and weak organic acid stress, Hsp30p limits ATP loss by regulating Pma1p activity [56, 57]. *STL1* encodes a glycerol/H^+^ symporter, which supports cell survival during high temperature and osmotic shock by fine-tuning the intracellular glycerol levels [58]. *AQR1* encodes a plasma membrane transporter belonging to the major facilitator superfamily (MFS), which is involved in multidrug resistance (MDR). It has been reported that *AQR1* confers resistance to short-chain monocarboxylic acids in *S. cerevisiae* [59]. The upregulation of these genes indicated that, in response to 20 g/L SA, cells might employ multiple strategies, including expulsing toxic compounds, limiting ATP consumption, and storing glycerol as protectant. Comparable mechanisms have been detected in *E. coli*, wherein the efficient expulsion of toxins and the accumulation of protective agents like betaine and proline allow the SA-tolerant *E. coli* strain to grow under severe SA stress [60].

For comparison 60 vs. 0, a total of 264 DEGs were identified (Fig. 2). Ten KEGG pathways related to thiamine and vitamin B6 metabolism, carbon metabolism, and amino acids metabolism were enriched (Table S3). In addition, DEGs were clustered into seven groups through PPI analysis, for instance, protein folding, protein targeting, thiamine metabolism, etc. (Fig. S3, Table S6). It seems that NBRC2018 responded to 60 g/L SA by regulating genes involved in the biosynthesis of vitamin B6 and thiamine, protein folding, and carbon metabolism (Fig. 5).

In response to 60 g/L SA, genes associated with the biosynthesis of vitamin B6 and thiamine were downregulated in NBRC2018, whereas most DEGs associated with protein folding were significantly upregulated. This result was consistent with that in NBRC1958 and KF7. However, a distinctive feature unique to NBRC2018 was the pronounced downregulation of the *HSP26* gene under 60 g/L SA. *HSP26*, together with *HSP42*, encodes the only members of the small heat shock proteins (sHsps) in *S. cerevisiae* [61]. Studies have demonstrated that sHsps co-aggregate with misfolded proteins and facilitate the refolding of protein aggregates [61, 62]. The decreased expression of *HSP26* in NBRC2018 might exacerbate the burden on the PQC system when encountering severe SA stress.

At high concentration of SA, there was also a notable impact on carbon metabolism in NBRC2018. Genes related to the PPP (*SOL4*, *TKL2*, and *NQM1*) and gluconeogenesis (*PYC1*, *PCK1* and *FBP1*) were significantly upregulated under 60 g/L SA. This suggests that SA might be diverted into gluconeogenesis to alleviate stress. In contrast to KF7, NBRC2018 uniquely displayed upregulation of certain genes necessary for the utilization of fatty acid and ethanol under high levels of SA. For example, transcription factors Adr1p and Nsf1p activate genes required for the utilization of ethanol, glycerol, and fatty acids; *ADH2* encodes alcohol dehydrogenase that converts ethanol to acetaldehyde; *POX1* and *POT1* play major roles in fatty acid beta-oxidation in peroxisome; and the carnitine transporter *CRC1* and the glyoxylate cycle genes (*CIT2*, *MLS1*, *IDP2*, and *ICL1*) are responsible for transferring peroxisome-derived acetyl-CoA to the mitochondria. The upregulation of these genes suggested that under 60 g/L SA, a substantial portion of the intracellular acetyl-CoA might be generated through the metabolism of ethanol and fatty acids. However, the elevated activity of the glyoxylate cycle could lead to increased production of endogenous succinate, which might further aggravate the intracellular SA stress.

In conclusion, NBRC2018 responds to 20 g/L SA by reducing ATP utilization, expelling toxins, and storing protectant like glycerol. When exposed to 60 g/L SA, the inhibition of cell growth became more severe. Although genes related to the PPP, gluconeogenesis, fatty acid utilization, ethanol utilization, and protein folding were significantly upregulated, the downregulation of *HSP26* and the overproduction of endogenous succinate due to the activation of the glyoxylate cycle likely intensified the SA stress. These observed transcriptional response mechanisms could, to a considerable extent, elucidate the poor tolerance of NBRC2018 cells to SA.

### Comparison of three strains with different genetic backgrounds

**Fig. 6:**
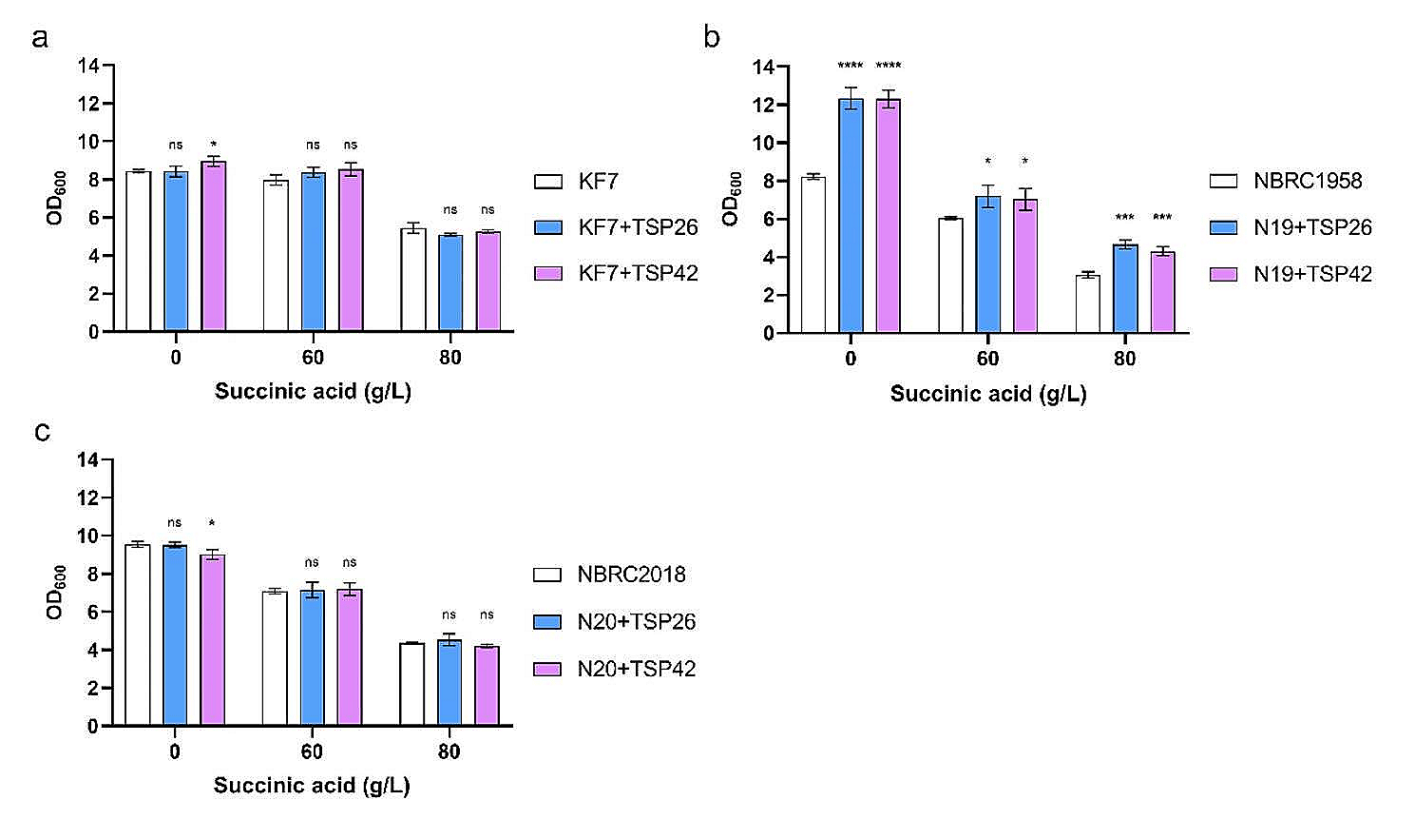
Influence of *HSP26* or *HSP42* overexpression on the SA tolerances of *S. cerevisiae* strains KF7 (a), NBRC1958 (b), and NBRC2018 (c). Cells were exposed to 0 g/L, 60 g/L, and 80 g/L SA, respectively. The initial inoculum was OD_600_ 0.4. OD_600_ was measured at 10 h of cultivation. Taking the original strains KF7, NBRC1958, and NBRC2018 as controls for each group. Values and standard deviations were calculated from three repeated samples. *p < 0.05; **p < 0.001; ***p < 0.0001; ****p < 0.00001; ns, no statistically significant difference

The overlapping responses of the three strains emphasized the importance of the PQC in coping with severe SA stress. PQC has been reported to address the misfolded proteins under diverse stresses like oxidation stress, heat shock, and ethanol exposure [32, 34, 63, 64]. An increased PQC activity might enhance the SA tolerance of *S. cerevisiae*. However, NBRC2018 showed a significant downregulation of *HSP26*, which could potentially undermine its ability to tolerate SA stress. Given that *HSP26* and *HSP42* encode the only two sHsps in *S. cerevisiae*, they might share similar functions in responding to SA stress. Therefore, to verify their roles in SA tolerance, we overexpressed *HSP26* and *HSP42* in all three strains. The constitutively strong promoter *TEF1*p was used to express *HSP26* and *HSP42*. Six strains were obtained and named as KF7 + TSP26, KF7 + TSP42, N19 + TSP26, N19 + TSP42, N20 + TSP26, and N20 + TSP42, respectively. The growths of these strains under different concentrations of SA (0 g/L, 60 g/L, and 80 g/L) were compared with their original strains (Fig. 6; Table 4).

**Table 4:** The growth enhancement ratios (%) of the engineered strains compared to its original strain under different concentrations of SA

SA	0 g/L	60 g/L	80 g/L
KF7 + TSP26 vs. KF7	-0.26	4.86	-6.49
KF7 + TSP42 vs. KF7	5.96	6.93	-3.19
N19 + TSP26 vs. NBRC1958	49.88	19.16	52.29
N19 + TSP42 vs. NBRC1958	49.39	16.26	40.68
N20 + TSP26 vs. NBRC2018	-0.42	0.94	4.01
N20 + TSP42 vs. NBRC2018	-5.75	1.51	-3.44

The growth enhancement ratio = (OD_600_ of the engineered strain - OD_600_ of its original strain)/ OD_600_ of its original strain. Positive values represent an increase in growth, while negative values represent a decrease in growth. The initial inoculum was OD_600_ 0.4. Data were calculated at 10 h of cultivation. Average values were calculated from three replicates

The overexpression of *HSP26* and *HSP42* led to a significant improvement in the growth of NBRC1958. In detail, when *HSP26* was overexpressed, the cell growth of NBRC1958 increased by 50%, 19%, and 52% at 0 g/L, 60 g/L, and 80 g/L SA, respectively. Overexpression of *HSP42* had a nearly identical effect on the cell growth of NBRC1958. The results indicated that enhancing the activity of sHsps can elevate the intrinsic growth capacity of NBRC1958, thus boosting its ability to grow under SA stress. This finding aligns with prior research, which demonstrated that sHsps serve as universal effectors of longevity, and the overexpression of *HSP26* extended the replicative lifespan of yeast cells [65]. Consistently, sHsps have been implicated in *S. cerevisiae*’s response to other weak acids, such as sorbic acid and citric acid [66, 67]. Overexpression of *HSP26* has been shown to enhance the strains’ tolerance to sorbic acid [66]. However, for the other two strains, neither the overexpression of *HSP26* nor *HSP42* had a notable effect on cell growth. It was likely that some other key limiting factors may play a more decisive role in determining the SA tolerance of the two strains. In conclusion, overexpressing *HSP26* or *HSP42* in strains with inherent low sHsps activities is one of the methods to improve SA tolerance.

Genetic background has been proven to affect sugar metabolism and inhibitor tolerance of *S. cerevisiae* [68, 69]. In this study, we observed that the response mechanisms of different strains to SA were indeed influenced by their genetic backgrounds (Figs. 3, 4 and 5). For example, KF7 and NBRC2018 showed notable differences in the regulation of acetyl-CoA metabolism under SA stress. When KF7 was exposed to SA, genes associated with fatty acids beta-oxidation and the glyoxylate cycle were significantly downregulated. As a result, the production of acetyl-CoA from peroxisomes reduced, leading to a correspondingly reduction in endogenous succinate synthesis. Instead, exogenous SA was likely to be used for acetyl-CoA biosynthesis to maintain intracellular acetyl-CoA levels in KF7 (Fig. 3). On the contrary, when NBRC2018 encountered severe SA stress (60 g/L), genes related to fatty acid β-oxidation and the glyoxylate cycle were significantly upregulated. Despite the increased availability of acetyl-CoA, this upregulation also promoted the generation of endogenous succinate, which further exacerbating the intracellular SA stress. This differential response may be one of the main reasons why NBRC2018 was less tolerant to SA than KF7. Thus, when developing SA-producing strains, it is crucial to carefully consider the intracellular acetyl-CoA levels and the strains’ capacity to utilize SA.

In general, weak acids typically cause toxicity in *S. cerevisiae* cells through several mechanisms, include intracellular acidification, membrane damage, oxidative stress, protein aggregation, carbon metabolism disruption, etc. [70]. Accordingly, yeast cells employed a variety of complex and diverse regulatory mechanisms to cope with weak acids [70, 71]. For instance, the activities of plasma membrane H^+^-ATPase and ABC transporters are increased in response to acetic acid [71]; the maintenance of CWI is crucial for yeast’s adaptation and tolerance to acetic and lactic acid [72]; the biosynthesis of purine and methionine is reduced in response to formic and acetic acids [73, 74]; the unfolded protein response (UPR) is induced by lactic, citric, and acetic acid [75, 76]; the synthesis of proline and glycerol is increased in response to oxidative stress triggered by lactic acid [77]; and spore formation is induced by formic and acetic acid stress [73, 74]. Furthermore, genes related to weak acid metabolism are upregulated to facilitate in situ detoxification of weak acids. For example, formic acid is oxidized into CO_2_ and H_2_O by the formate dehydrogenase [74]; lactate is converted to pyruvate by lactate dehydrogenase [78]; citrate can be utilized as an energy source via the TCA cycle [67]; acetate is directly converted to acetyl-CoA, which enter either the TCA cycle or gluconeogenesis [75]. The responses of *S. cerevisiae* to SA observed in this study encompass all these aspects. Notably, a unique response is the potential conversion of excess SA to malate, which then enters gluconeogenesis. The finding is consistent with previous study [79]. Additionally, excess SA may prevent the formation of endogenous succinate from the glyoxylate cycle (Fig. 3).

In addition to weak acids, some recent reports have focused on various stress-resistant *S. cerevisiae* strains obtained through evolutionary engineering, along with investigations into their adaptive resistance mechanisms [80–83]. The findings revealed that when exposed to AgNPs, the silver-resistant strain 2E showed a significant downregulation of mannoprotein genes (*TIP1*, *TIR1-4*, *RNT1*, *YVH1*) and an upregulation of other cell wall-associated genes like *YPK2*, *USV1*, *YPS6*, and *SRL1* [80]. In the case of 2-phenylethanol (2-PE) stress, the modified cell wall structure and increased expulsion of 2-PE from the cell potentially explained the 2-PE resistance in the evolved strain C9 [81]. Under caffeine stress, the caffeine-tolerant strain Caf905-2 exhibited increased expression of genes related to glycolysis, the PPP (*SOL4* and *GND2*), protein folding, toxin efflux (*PDR1* and *PDR5*), and cell wall integrity (*RIM8*) [82]. When subjected to oxidative stress, genes related to cell wall organization, protein folding (*HSP26*), transmembrane transport, sporulation, and the stationary phase were strongly upregulated in the oxidative stress-resistant strain H7 [83]. These studies, along with the results of the present study, collectively highlight the significance of maintaining both the structural integrity and functional performance of cell membranes and cell walls, as well as enhanced protein folding, in coping with a wide range of stresses.

## Conclusion

In this study, five *S. cerevisiae* strains with different genetic backgrounds were compared for their SA tolerances. KF7 and NBRC1958 with excellent SA tolerances, and NBRC2018 with poor SA tolerance, were selected to investigate the response mechanisms of *S. cerevisiae* to SA through comparative transcriptomic analysis. Few genes were significantly regulated under 20 g/L SA in three strains. When exposed to 60 g/L SA, the three strains showed different response mechanisms. Overall, the DEGs were involved in carbon metabolism, amino acid metabolism, protein folding, meiosis, membrane proteins and cell wall structure. We conclude that the genetic background of the host strain is important for the construction of good SA producing strains. The inherent activities of sHsps, acetyl-CoA levels and the potential SA consumption capacity of the host strains must be considered. This study provides theoretical guidance and tolerant strains for the breeding of robust SA-producing strains.

### Electronic supplementary material

Below is the link to the electronic supplementary material.

Supplementary Material 1

Supplementary Material 2

## Data Availability

The dataset(s) used and/or analyzed during the current study are available from the corresponding author on reasonable request. The original microarray data can be accessed in the National Center for Biotechnology Information (http://www.ncbi.nlm.nih.gov/) through GEO accession GSE193190.

## Abbreviations

*S. cerevisiae*: *Saccharomyces cerevisiae*
SA: Succinic acid
PPP: Pentose phosphate pathway
NADPH: Nicotinamide adenine dinucleotide phosphate
PQC: Protein quality control
sHsps: Small heat-shock proteins
*E*. *coli*: *Escherichia coli*
DEGs: Differentially expressed genes
FPKM: Fragments per kilobase of exon per million reads mapped
FC: Fold change
DNA: Deoxyribonucleic acid
RNA: Ribonucleic Acid
KEGG: Kyoto Encyclopedia of Genes and Genomes
PPI: Protein-protein interaction
acetyl-CoA: Acetyl coenzyme A
TCA cycle: Tricarboxylic acid cycle
VB6: Vitamin B6
DSB: DNA double-strand breaks
PLP: Pyridoxal 5′-phosphate
TPP: Thiamine pyrophosphate
cAMP: Cyclic AMP
PKA: Protein kinase A
CWI: Cell wall integrity
ATP: Adenosine-triphosphate
ABC: ATP-binding cassette
PDR: Pleiotropic drug resistance
GID: Glucose-induced degradation
MFS: Major facilitator superfamily
MDR: Multidrug resistance
UPR: Unfolded protein response
LB: Luria-Bertani
OD_600_: Absorbance at 600 nm
SGD: Saccharomyces Genome Database
2-PE: 2-phenylethanol
