# Supplementary material for: Response mechanisms of different Saccharomyces cerevisiae strains to succinic acid
Source: BMC Microbiol. 2024 May 8;24:158. doi: 10.1186/s12866-024-03314-4 (PMC11077785; doi:10.1186/s12866-024-03314-4)
Supplement: Supplementary file 1 — Supplementary Material 1 [file 12866_2024_3314_MOESM1_ESM.docx]

Supplementary materials 1

Tile: Response mechanisms of different *Saccharomyces cerevisiae* strains to succinic acid

Authors:

Cai-Yun Xie^1, 2, 4^, Ran-Ran Su^1, 2^, Bo Wu^3^, Zhao-Yong Sun^1,2,4^, Yue-Qin Tang^1, 2, 4^*

Affiliation:

^1^College of Architecture and Environment, Sichuan University, No. 24 South Section 1 First Ring Road, Chengdu 610065, Sichuan, China

^2^Sichuan Environmental Protection Key Laboratory of Organic Wastes Valorization, No. 24 South Section 1 First Ring Road, Chengdu 610065, Sichuan, China

^3^Biogas Institute of Ministry of Agriculture, Renmin Rd. 4-13, Chengdu 610041, Sichuan, China

^4^Engineering Research Center of Alternative Energy Materials & Devices, Ministry of Education, No. 24 South Section 1 First Ring Road, Chengdu 610065, Sichuan, China

* Corresponding author:

Tel. (fax): +86 2885990936;

Email address: [tangyq@scu.edu.cn](mailto:tangyq@scu.edu.cn)

Other authors’ Email address:

xiecy@scu.edu.cn; [surr0806@163.com](mailto:surr0806@163.com); [wubo@caas.cn](mailto:wubo@caas.cn); [szy@scu.edu.cn](mailto:szy@scu.edu.cn).

**Table S1** FPKM values of genes under different concentrations of SA.

| Strains | Genes | 0 g/L SA | 20 g/L SA | 60 g/L SA |
| --- | --- | --- | --- | --- |
| KF7 | *HSP26* | 2517.16 | 1480.13 | 3915.67 |
|  | *HSP42* | 3180.95 | 2510.01 | 6141.57 |
|  | *TEF1* | 7165.17 | 6924.43 | 7187.51 |
|  | *YJL043W* | 12.18 | 14.74 | 14.74 |
| NBRC1958 | *HSP26* | 1575.74 | 669.71 | 2342.19 |
|  | *HSP42* | 4278.51 | 4827.74 | 6876.81 |
|  | *TEF1* | 7068.79 | 7102.63 | 7392.15 |
|  | *YJL043W* | 15.74 | 14.74 | 14.74 |
| NBRC2018 | *HSP26* | 5953.87 | 5975.96 | 2602.97 |
|  | *HSP42* | 2092.41 | 3000.09 | 4489.41 |
|  | *TEF1* | 6887.84 | 7093.05 | 7102.63 |
|  | *YJL043W* | 17.65 | 16.06 | 14.74 |

**Table S2** Sequence of the *TEF1* promoter.

| Name | Sequence（5’~3’） |
| --- | --- |
| P*_TEF1_* | CAGAAAGCGACCACCCAACTTTGGCTGATAATAGCGTATAAACAATGCATACTTTGTACGTTCAAAATACAATGCAGTAGATATATTTATGCATATTACATATAATACATATCACATAGGAAGCAACAGGCGCGTTGGACTTTTAATTTTCGAGGACCGCGAATCCTTACATCACACCCAATCCCCCACAAGTGATCCCCCACACACCATAGCTTCAAAATGTTTCTACTCCTTTTTTACTCTTCCAGATTTTCTCGGACTCCGCGCATCGCCGTACCACTTCAAAACACCCAAGCACAGCATACTAAATTTCCCCTCTTTCTTCCTCTAGGGTGTCGTTAATTACCCGTACTAAAGGTTTGGAAAAGAAAAAAGAGACCGCCTCGTTTCTTTTTCTTCGTCGAAAAAGGCAATAAAAATTTTTATCACGTTTCTTTTTCTTGAAAATTTTTTTTTTTGATTTTTTTCTCTTTCGATGACCTCCCATTGATATTTAAGTTAATAAACGGTCTTCAATTTCTCAAGTTTCAGTTTCATTTTTCTTGTTCTATTACAACTTTTTTTACTTCTTGCTCATTAGAAAGAAAGCATAGCAATCTAATCTAAGTTTTAATTACAAA |

**Table S3** Enriched KEGG pathways (*p* <0.005).

| KEGG Pathway | Description | Genes |
| --- | --- | --- |
| KF7 (20 *vs.* 0) | |  |
| sce04146 | Peroxisome | *AGX1, IDP2, YAT1, POT1, YAT2* |
| KF7 (60 *vs.* 0) | |  |
| sce01130 | Biosynthesis of antibiotics | *ALD6, INO1, MET2, MET3, SER3, GCV1, GCV3, GCV2, ADE17, PCK1, CTA1, IDP2, CAR2, SOL4, CAR1, NQM1, AAT1, ARO9, CHA1, YHR033W, ADE4, ADE1, IRC15, GND2, PYK2, POT1, TKL2, SHM2* |
| sce01100 | Metabolic pathways | *MAL12, INO1, FDH1, HST4, HST3, LEU2, ADE17, MHT1, IDP2, NQM1, ARO9, GDH2, GDH3, CHA1, IMD2, GND2, PYK2, DAL7, CYB2, POT1, TKL2, DAL3, DAL2, SHM2, DAL1, SGA1, ALD6, BNA1, MAL32, MET2, MET3, BIO2, SER3, GCV1, GCV3, COX11, GCV2, PCK1, ICL1, CAR2, SOL4, CAR1, AAT1, STR3, ARO10, YHR033W, ADE4, MTD1, ADE1, COQ2, IRC15* |
| sce01110 | Biosynthesis of secondary metabolites | *ALD6, LEU2, GCV1, GCV3, GCV2, ADE17, PCK1, MHT1, ICL1, CTA1, IDP2, CAR2, SOL4, CAR1, NQM1, AAT1, ARO9, STR3, CHA1, ADE4, IMD2, ADE1, COQ2, IRC15, GND2, PYK2, DAL7, POT1, TKL2, SHM2* |
| sce01200 | Carbon metabolism | *SOL4, NQM1, AAT1, FDH1, CHA1, IRC15, GND2, SER3, DAL7, TKL2, PCK1, CTA1, PYK2, GCV1, GCV2, SHM2, ICL1, IDP2* |
| sce00630 | Glyoxylate and dicarboxylate metabolism | *CTA1, FDH1, DAL7, IRC15, ICL1, SHM2, GCV3, GCV1, GCV2* |
| sce00750 | Vitamin B6 metabolism | *SNO1, SNO3, SNZ3, SNZ1, SNO2, SNZ2* |
| sce00620 | Pyruvate metabolism | *PCK1, GRE2, DAL7, IRC15, ACH1, PYK2, CYB2, ALD6* |
| sce00330 | Arginine and proline metabolism | *CAR2, AMD2, AAT1, CAR1, YHR033W, ALD6* |
| sce00260 | Glycine, serine and threonine metabolism | *CHA1, IRC15, SER3, SHM2, GCV3, GCV1, GCV2* |
| NBRC1958 (20 *vs.* 0) | |  |
| sce00730 | Thiamine metabolism | *THI5, THI13, THI4, THI11, THI12, THI21* |
| sce01100 | Metabolic pathways | *ARG4, PDC5, STR3, MET10, THI5, YHR033W, INM1, MET3, THI11, THI21, THI13, SUC2, THI12, THI4, MHT1, MET6, MET17* |
| sce00750 | Vitamin B6 metabolism | *SNO3, SNZ3, SNO2, SNZ2* |
| NBRC1958 (60 *vs.* 0) | |  |
| sce01100 | Metabolic pathways | *MET14, PUT1, FDH1, HST4, HST3, RPC11, ADE12, THI20, THI21, IMA1, PGU1, ADE13, ADE17, MHT1, CAT5, ARG4, ILV6, CPA1, HIS4, ARG3, ARO9, ARG1, GPT2, THI6, THI5, ARO2, NMA1, GPI12, RMA1, ETR1, PRI1, IMD2, IMD3, HMG2, POT1, TKL2, DAL3, SHM2, DAL1, MET17, BAT1, BNA1, INM2, URA1, THI80, ADH5, MET3, GCV1, FLO11, THI4, GCV3, UTR1, GCV2, YDR248C, PCK1, ICL1, MET5, MET6, CAR1, PDC6, PDC5, AAT1, PRS2, STR3, MDH2, ARO10, SOL1, YHR033W, ADE4, ADE2, CKI1, MTD1, ADE1, IRC15, THI11, THI13, THI12, CEM1, GUD1* |
| sce00730 | Thiamine metabolism | *THI5, THI13, THI4, THI11, THI20, THI80, THI12, THI21, THI6* |
| sce01110 | Biosynthesis of secondary metabolites | *BAT1, PUT1, ADH5, GCV1, ADE13, GCV3, GCV2, ADE17, YDR248C, PCK1, MHT1, ICL1, CTA1, MET6, ARG4, CAT5, ILV6, CAR1, PDC6, PDC5, HIS4, ARG3, AAT1, PRS2, ARO9, ARG1, STR3, MDH2, GPT2, SOL1, ARO2, ADE4, ADE2, IMD2, ADE1, IMD3, HMG2, IRC15, POT1, TKL2, SHM2, MET17* |
| sce01130 | Biosynthesis of antibiotics | *BAT1, PUT1, ADH5, MET3, GCV1, ADE13, GCV3, GCV2, ADE17, YDR248C, PCK1, CTA1, ARG4, ILV6, CAR1, ARG3, AAT1, PRS2, ARO9, ARG1, MDH2, SOL1, ARO2, YHR033W, ADE4, ADE1, HMG2, IRC15, POT1, TKL2, SHM2, MET17* |
| sce00630 | Glyoxylate and dicarboxylate metabolism | *CTA1, MDH2, FDH1, IRC15, ICL1, SHM2, GCV3, GCV1, GCV2* |
| sce00230 | Purine metabolism | *RPC11, PDE2, DAL1, DAL3, ADE13, IMD3, ADE1, GUD1, MET3, MET14, ADE2, PRI1, ADE17, ADE4, ADE12, IMD2, PRS2* |
| NBRC2018 (20 *vs.* 0) | |  |
| sce01130 | Biosynthesis of antibiotics | *CIT2, PCK1, YHR033W, PUT1* |
| NBRC2018 (60 *vs.* 0) | |  |
| sce01100 | Metabolic pathways | *MAL12, INO1, TSC10, FDH1, YPC1, RPA14, THI20, IMA3, THI21, IMA4, IMA1, SUC2, IMA2, IRC7, IDP2, CAT5, NQM1, THI6, THI5, RPA43, IMD2, PYC1, MLS1, POT1, TKL2, SHM2, FBP1, POX1, MET17, SGA1, BAT2, BNA1, ALD3, THI80, MAL32, ADH2, MET3, SER3, GCV1, THI4, GCV3, GCV2, CIT2, PCK1, ICL1, MET6, SOL4, CAR1, PDC6, PDC5, IDH1, YHR033W, ADE4, CKI1, MTD1, ADE1, IRC15, THI11, THI13, THI12, CEM1, BUD16* |
| sce00730 | Thiamine metabolism | *THI5, THI13, PHO3, THI4, THI11, THI20, THI80, THI12, THI21, THI6* |
| sce00630 | Glyoxylate and dicarboxylate metabolism | *CIT2, CTA1, CTT1, FDH1, MLS1, IRC15, ICL1, SHM2, GCV2, GCV3, GCV1* |
| sce01200 | Carbon metabolism | *CIT2, IDP2, FBP1, CTA1, TKL2, PYC1, NQM1, CTT1, SOL4, PCK1, FDH1, MLS1, IDH1, IRC15, ICL1, SER3, SHM2, MET17, GCV1, GCV2* |
| sce01110 | Biosynthesis of secondary metabolites | *BAT2, ALD3, CTT1, ADH2, GCV1, GCV3, GCV2, CIT2, PCK1, ICL1, CTA1, MET6, IRC7, IDP2, CAT5, SOL4, CAR1, PDC6, PDC5, NQM1, IDH1, ADE4, IMD2, ADE1, IRC15, MLS1, POT1, TKL2, SHM2, FBP1, POX1, MET17* |
| sce01130 | Biosynthesis of antibiotics | *BAT2, INO1, CTT1, MET3, ADH2, SER3, GCV1, GCV3, GCV2, CIT2, PCK1, CTA1, IDP2, SOL4, CAR1, NQM1, IDH1, YHR033W, ADE4, ADE1, IRC15, POT1, TKL2, SHM2, FBP1, MET17* |
| sce00052 | Galactose metabolism | *MAL32, IMA1, MAL12, IMA4, IMA3, IMA2, SUC2* |
| sce00500 | Starch and sucrose metabolism | *MAL32, IMA1, MAL12, IMA4, IMA3, IMA2, SUC2, SGA1* |
| sce01230 | Biosynthesis of amino acids | *CIT2, IDP2, TKL2, PYC1, NQM1, IDH1, CAR1, YHR033W, SER3, MET6, IRC7, MET17, BAT2, SHM2* |
| sce00750 | Vitamin B6 metabolism | *SNO2, SNZ3, SNO3, SNZ2, BUD16* |

Red and green colors represent upregulated and downregulated genes, respectively.

**Table S4** Protein-protein interaction groups of KF7 for DEGs between the SA (60 g/L) and the control group. DEGs involved in each group were shown.

| Gene name | Description | Fold change |
| --- | --- | --- |
| Protein folding |  |  |
| *SSA4* | Heat shock protein | 13.30 |
| *APJ1* | Chaperone with a role in SUMO-mediated protein degradation | 8.20 |
| *HSP78* | Oligomeric mitochondrial matrix chaperone | 5.82 |
| *SIS1* | Type II HSP40 co-chaperone | 3.44 |
| *HSC82* | Cytoplasmic chaperone of the Hsp90 family | 3.18 |
| *HSP82* | Hsp90 chaperone | 3.18 |
| *SSA3* | ATPase | 2.90 |
| *STI1* | Hsp90 cochaperone | 2.59 |
| *SSA2* | ATP-binding protein | 2.50 |
| *SSA1* | ATPase | 2.46 |
| *MDJ1* | Co-chaperone | 2.08 |
| VB6 |  |  |
| *SNO1* | Involved in pyridoxine metabolism | -5.99 |
| *SNZ1* | Protein involved in vitamin B6 biosynthesis | -5.20 |
| *SNZ2* | GMP synthase activity | -2.70 |
| *SNZ3* | Pyridoxal-5′-phosphate synthase | -2.70 |
| *SNO2//SNO3* | Putative pyridoxal 5′-phosphate synthase | -2.48 |
| *SNO4//HSP32//SHP33* | Possible chaperone and cysteine protease | 2.29 |
| Meiotic |  |  |
| *MMS4* | Subunit of endonuclease | 3.71 |
| *SPO11* | Meiosis-specific protein | 2.66 |
| *SPO13* | Meiotic regulator | 2.48 |
| *RDH54* | DNA-dependent ATPase | 2.24 |
| Amino acid metabolic process |  |  |
| *GCV1* | T subunit of the mitochondrial glycine decarboxylase complex | -4.42 |
| *GCV2* | P subunit of the mitochondrial glycine decarboxylase complex | -3.17 |
| *GCV3* | H subunit of the mitochondrial glycine decarboxylase complex | -2.06 |
| *IRC15* | Microtubule associated protein | 5.56 |
| *GDH3* | NADP(+)-dependent glutamate dehydrogenase | 2.98 |
| *GDH2* | NAD(+)-dependent glutamate dehydrogenase | 2.15 |

**Table S5** Protein-protein interaction groups of NBRC1958 for DEGs between the SA (60 g/L) and the control group. DEGs involved in each group were shown.

| Gene name | Description | Fold change |
| --- | --- | --- |
| Response to chemical |  |  |
| *PDE2* | High-affinity cyclic AMP phosphodiesterase | 2.74 |
| *RAD6* | Ubiquitin-conjugating enzyme | 2.12 |
| *IRA2* | GTPase-activating protein | 2.02 |
| *IMD2* | Inosine monophosphate dehydrogenase | -6.01 |
| *HAP2* | Subunit of the Hap2p/3p/4p/5p CCAAT-binding complex | -3.28 |
| *ADE17* | Enzyme of 'de novo' purine biosynthesis | -2.48 |
| *TPK2* | cAMP-dependent protein kinase catalytic subunit | -2.44 |
| *TSA2* | Stress inducible cytoplasmic thioredoxin peroxidase | -2.20 |
| *ADE12* | Adenylosuccinate synthase | -2.16 |
| *PPG1* | Putative serine/threonine protein phosphatase | -2.10 |
| *TRX3* | Mitochondrial thioredoxin | -2.09 |
| *EPS1* | ER protein with chaperone and co-chaperone activity | -2.07 |
| *HAP5* | Subunit of the Hap2p/3p/4p/5p CCAAT-binding complex | -2.05 |
| *MSN4* | Stress-responsive transcriptional activator | -2.00 |
| Protein folding |  |  |
| *SSA4* | Heat shock protein | 8.18 |
| *APJ1* | Chaperone with a role in SUMO-mediated protein degradation | 6.63 |
| *HSP82* | Hsp90 chaperone | 4.35 |
| *HSP78* | Oligomeric mitochondrial matrix chaperone | 3.69 |
| *SSA2* | ATP-binding protein | 3.41 |
| *STI1* | Hsp90 cochaperone | 3.29 |
| *SLP1* | Glycosylated integral ER membrane protein | 2.63 |
| *HSC82* | Cytoplasmic chaperone of the Hsp90 family | 2.61 |
| *SSA3* | ATPase | 2.58 |
| *KAR2* | ATPase involved in protein import into the ER | 2.29 |
| *SSA1* | ATPase | 2.27 |
| *SSE1* | ATPase component of heat shock protein Hsp90 chaperone complex | 2.18 |
| *YDJ1* | Type I HSP40 co-chaperone | 2.14 |
| Nicotinate and nicotinamide metabolism |  |  |
| *PDR5* | Plasma membrane ATP-binding cassette (ABC) transporter | 3.64 |
| *TBS1* | Putative protein of unknown function | 3.26 |
| *WSC3* | Sensor-transducer of the stress-activated PKC1-MPK1 signaling pathway | 3.17 |
| *MID2* | O-glycosylated plasma membrane protein | 2.93 |
| *ROM1* | GDP/GTP exchange protein (GEP) for Rho1p | 2.70 |
| *SNQ2* | Plasma membrane ATP-binding cassette (ABC) transporter | 2.59 |
| *HST4* | Member of the Sir2 family of NAD(+)-dependent protein deacetylases | 2.56 |
| *NMA1* | Nicotinic acid mononucleotide adenylyltransferase | 2.34 |
| *HST3* | Member of the Sir2 family of NAD(+)-dependent protein deacetylases | 2.26 |
| *PDR12* | Plasma membrane ATP-binding cassette (ABC) transporter | 2.04 |
| *UTR1* | ATP-NADH kinase | -2.27 |
| Monosaccharide transmembrane transporter activity |  |  |
| *HXT5* | Hexose transporter with moderate affinity for glucose | 6.41 |
| *HXT1* | Low-affinity glucose transporter of the major facilitator superfamily | 5.14 |
| *HXT2* | High-affinity glucose transporter of the major facilitator superfamily | 4.74 |
| *CPR6* | Protein abundance increases in response to DNA replication stress | 2.83 |
| *PRP46* | Stabilizes U6 snRNA | 2.66 |
| *GAL2* | Galactose permease; required for utilization of galactose; able to transport glucose | 2.31 |
| *CUS1* | Protein required for assembly of U2 snRNP into the spliceosome | 2.24 |
| *HSH49* | U2-snRNP associated splicing factor | 2.19 |
| *PRP4* | Splicing factor | -2.28 |
| *PRP21* | Required for spliceosome assembly | -2.09 |
| Glyoxylate and dicarboxylate metabolism |  |  |
| *IRC15* | Required for accurate meiotic chromosome segregation | 7.90 |
| *CTF19* | Needed for accurate chromosome segregation | 3.05 |
| *BNR1* | Formin; nucleates the formation of linear actin filaments | 2.87 |
| *MCM16* | Involved in kinetochore-microtubule mediated chromosome segregation | 2.68 |
| *SPC24* | Involved in chromosome segregation, spindle checkpoint activity, and kinetochore clustering | 2.21 |
| *GCV1* | Required for the catabolism of glycine to 5, 10-methylene-THF | -5.92 |
| *MTD1* | Plays a catalytic role in oxidation of cytoplasmic one-carbon units | -5.00 |
| *GCV2* | Required for the catabolism of glycine to 5, 10-methylene-THF | -3.55 |
| *SHM2* | Major isoform involved in generating precursors for purine, pyrimidine, amino acid, and lipid biosynthesis | -3.23 |
| *GCV3* | Required for the catabolism of glycine to 5, 10-methylene-THF | -2.35 |
| Amino acid metabolic process |  |  |
| *PDC6* | Minor isoform of pyruvate decarboxylase, strongly induced during sulfur limitation | 2.58 |
| *AAT1* | Mitochondrial aspartate aminotransferase | 2.58 |
| *ARO9* | Aromatic aminotransferase II; catalyzes the first step of tryptophan, phenylalanine, and tyrosine catabolism | 2.08 |
| *PDC5* | Minor isoform of pyruvate decarboxylase; repressed by thiamine, involved in amino acid catabolism | -6.64 |
| *MET17* | Required for Methionine and cysteine biosynthesis | -5.66 |
| *ARO10* | Phenylpyruvate decarboxylase, involved in protein N-terminal Met and Ala catabolism | -4.36 |
| *MET6* | Involved in methionine biosynthesis and regeneration | -4.15 |
| *STR3* | Converts cystathionine into homocysteine | -2.67 |
| *MHT1* | S-methylmethionine-homocysteine methyltransferase | -2.55 |
| *BAT1* | Mitochondrial branched-chain amino acid (BCAA) aminotransferase, highly expressed during logarithmic phase and repressed during stationary phase | -2.21 |
| Transmembrane transporter activity |  |  |
| *MUP1* | High affinity methionine permease | 5.10 |
| *MEP1* | Ammonium permease | 3.81 |
| *PRR2* | Serine/threonine protein kinase | 3.65 |
| *MEP2* | Ammonium permease involved in regulation of pseudohyphal growth | 2.42 |
| *URA1* | Catalyzes the fourth enzymatic step in the de novo biosynthesis of pyrimidines | 2.15 |
| *FUR4* | Plasma membrane localized uracil permease | 2.10 |
| *DAL1* | Expression sensitive to nitrogen catabolite repression | 2.04 |
| *YAR028W* | Putative integral membrane protein | -2.19 |
| *FUI1* | High affinity uridine permease, localizes to the plasma membrane | -2.07 |

**Table S6** Protein-protein interaction groups of NBRC2018 for DEGs between the SA (60 g/L) and the control group. DEGs involved in each group were shown.

| Gene name | Description | Fold change |
| --- | --- | --- |
| Protein folding |  |  |
| *HSP26* | Small heat shock protein (sHSP) with chaperone activity | -2.29 |
| *HSP42* | Small heat shock protein (sHSP) with chaperone activity | 2.15 |
| *CUR1* | Sorting factor, central regulator of spatial protein quality control | 3.11 |
| *BTN2* | v-SNARE binding protein | 15.48 |
| *HSP104* | Disaggregase, heat shock protein that cooperates with Ydj1p (Hsp40) and Ssa1p (Hsp70) to refold and reactivate previously denatured, aggregated proteins | 2.54 |
| *APJ1* | Chaperone with a role in SUMO-mediated protein degradation | 7.93 |
| *CIT2* | Citrate synthase | 3.53 |
| *HSP78* | Oligomeric mitochondrial matrix chaperone | 4.69 |
| *MLS1* | Malate synthase, enzyme of the glyoxylate cycle | 3.87 |
| *PCK1* | Phosphoenolpyruvate carboxykinase | 11.88 |
| *PYC1* | Pyruvate carboxylase isoform | 2.14 |
| *FDH1* | NAD(+)-dependent formate dehydrogenase | 11.42 |
| Biosynthesis of secondary metabolites |  |  |
| *TKL2* | Transketolase | 2.47 |
| *NQM1* | Transaldolase of unknown function | 2.00 |
| *PDC5* | Minor isoform of pyruvate decarboxylase | -2.63 |
| *PDC6* | Minor isoform of pyruvate decarboxylase | 2.44 |
| *ALD3* | Cytoplasmic aldehyde dehydrogenase | 2.42 |
| *ADH2* | Glucose-repressible alcohol dehydrogenase II | 5.62 |
| Cellular carbohydrate catabolic process |  |  |
| *SGA1* | Intracellular sporulation-specific glucoamylase | 2.86 |
| *MAL32* | Maltase (alpha-D-glucosidase) | 4.20 |
| *MAL12* | Maltase (alpha-D-glucosidase) | 4.20 |
| *SUC2* | Invertase | 2.39 |
| Organic hydroxy compound biosynthetic process |  |  |
| *HSP33* | Possible chaperone and cysteine protease | 2.91 |
| *HSP32* | Possible chaperone and cysteine protease | 2.91 |
| *SNO4* | Possible chaperone and cysteine protease | 2.91 |
| *SNO2* | Protein of unknown function | -7.49 |
| *SNZ3* | Member of a stationary phase-induced gene family | -10.35 |
| *SNZ2* | Member of a stationary phase-induced gene family | -10.35 |
| *BUD16* | Putative pyridoxal kinase | -2.12 |
| *SNO3* | Protein of unknown function | -7.49 |
| One-carbon metabolic process |  |  |
| *GCV1* | T subunit of the mitochondrial glycine decarboxylase complex | -7.04 |
| *GCV2* | P subunit of the mitochondrial glycine decarboxylase complex | -7.89 |
| *GCV3* | H subunit of the mitochondrial glycine decarboxylase complex | -2.71 |
| *MTD1* | NAD-dependent 5, 10-methylenetetrahydrafolate dehydrogenase | -4.18 |
| *IRC15* | Microtubule associated protein | 6.20 |
| *SHM2* | Cytosolic serine hydroxymethyltransferase | -3.19 |
| Thiamine metabolism |  |  |
| *THI80* | Thiamine pyrophosphokinase; | -2.36 |
| *THI20* | Trifunctional enzyme of thiamine biosynthesis, degradation and salvage | -4.60 |
| *THI21* | Hydroxymethylpyrimidine (HMP) and HMP-phosphate kinase | -7.97 |
| *THI6* | Thiamine-phosphate diphosphorylase and hydroxyethylthiazole kinase; required for thiamine biosynthesis | -2.89 |
| *PHO3* | Constitutively expressed acid phosphatase similar to Pho5p | -3.58 |
| Protein targeting |  |  |
| *SSA4* | Heat shock protein that is highly induced upon stress | 3.43 |
| *STI1* | Hsp90 cochaperone | 2.32 |
| *HSP82* | Hsp90 chaperone | 3.86 |

Fig. S1 Protein-protein interaction analysis of DEGs was carried out for comparisons KF7 20 *vs.* 0 (a) and KF7 60 *vs.* 0(b), respectively. DEGs involved in each protein interaction group were shown.

Fig. S2 Protein-protein interaction analysis of DEGs was carried out for comparisons NBRC1958 20 *vs.* 0 (a) and NBRC1958 60 *vs.* 0 (b), respectively. DEGs involved in each protein interaction group were shown.

Fig. S3 Protein-protein interaction analysis of DEGs was carried out for comparison NBRC2018 60 *vs.* 0. DEGs involved in each protein interaction group were shown.
